# Multiple Spontaneous Vertebral Fractures in a Younger Post-menopausal Woman Upon Stopping Denosumab Therapy

**DOI:** 10.1210/jcemcr/luad042

**Published:** 2023-05-17

**Authors:** Xun Yang Hu, William D Leslie, Gregory Kline

**Affiliations:** Cumming School of Medicine, University of Calgary, Calgary, AB T2T 5C7, Canada; Max Rady College of Medicine, University of Manitoba, Winnipeg, MB R2H 2A6, Canada; Cumming School of Medicine, University of Calgary, Calgary, AB T2T 5C7, Canada

**Keywords:** bisphosphonate, osteoporosis, vertebral fracture, denosumab

## Abstract

Denosumab is a widely used medication for the treatment of osteoporosis. It has been observed in recent years that abruptly stopping denosumab leads to an increase in bone turnover markers, a decrease in bone mineral density, and a higher incidence of vertebral fractures. We present the case of a 53-year-old woman with few comorbidities and no prior fragility fractures who experienced 4 spontaneous and severely debilitating vertebral fractures 5-months post denosumab discontinuation. At the time of her fractures, she was found to have markedly elevated bone turnover markers, despite bone mineral density that was not significantly changed from measurements done while on denosumab treatment. She went on to be treated with an alternative antiresorptive agent, risedronate, and had substantial declines in her bone turnover markers, along with clinical improvement in her back pain. She experienced no further fractures while on treatment. Abrupt discontinuation of denosumab without starting an alternative antiresorptive agent can lead to spontaneous vertebral fractures. These fractures can occur in young patients with no prior history of fragility fractures and can be severely debilitating. An alternative antiresorptive agent should be started in the case of denosumab discontinuation.

## Introduction

### Denosumab Mechanism of Action

Denosumab is a fully human monoclonal antibody that binds to the cytokine receptor activator of nuclear factor kappa B ligand with high affinity and inhibits osteoclast maturation, function, and survival, resulting in reduced bone resorption and increased bone mineral density [1]. It is given every 6 months through subcutaneous injection and is cleared by the reticuloendothelial system with a half-life of roughly 26 days. Denosumab is a treatment of osteoporosis, and the FREEDOM Extension trial supports its use for up to 10 years, which shows continued gains in bone mineral density (BMD) and sustained antifracture efficacy [2].

### Denosumab Discontinuation-rebound

Patients on antiresorptive therapies typically have their BMD reassessed at 3 to 5-years on therapy. For patients using bisphosphonates, a drug holiday may be considered for those with favorable risk profiles. However, it has been consistently observed in retrospective and prospective studies that starting 6 months after the last injection of denosumab, BMD quickly decreases back to pretreatment levels by as early as 9 months [3, 4]. The degree of BMD loss varies between studies, but all the gains obtained from treatment can be lost over the next 36 months [3, 4].

Multiple case reports and retrospective case series have been published that detail the occurrence of vertebral fractures as early as 7 months after denosumab discontinuation, with a vertebral fracture rate up to 7.1 per 100 patient-years (vs 1.2 per 100 patient-years while on treatment and 8.5 per 100 patient-years in placebo-treated women) [3]. However, among those with vertebral fractures, 60.7% in the denosumab-stopping group were multiple fractures vs 38.7% in the placebo patients (*P* = .049) [3]. Additionally, the annual percentage loss in BMD was highest for those with multiple vertebral fractures, intermediate for those with single vertebral fractures, and lowest for those with no vertebral fractures (−3.5% per year vs −2.2% per year vs −1.9% per year) [3]. Reports of multiple spontaneous vertebral fractures, especially in younger low-risk women such as the case presented here, point toward a unique denosumab-discontinuation relationship. The existing evidence has been considered sufficient to prompt expert guidelines that recommend starting a bisphosphonate with denosumab discontinuation [5].

We present a case of a 53-year-old woman who experienced 4 spontaneous vertebral fractures after discontinuation of denosumab and the use of bisphosphonates as treatment and secondary prevention. Her case is a reminder that these fractures can occur in a younger woman with few comorbidities, and, rather than presenting with incidental x-ray findings, she struggled with life-limiting pain and disabilities for the year after her incident. This underscores the potentially severe nature of denosumab-related fractures and the clinical impact they can have on people's lives.

## Case Presentation

A high-functioning 53-year-old woman was referred for assessment to an osteoporosis clinic after the occurrence of spontaneous, multiple vertebral fractures. There was no history of previous fragility fractures and no family history of fractures. She was a nonsmoker and did not use alcohol. Her diet contained 3 servings of dairy per day, and she took 400 IU of vitamin D and 650 mg of calcium daily as supplements. Her past medical history also included hypothyroidism, for which she took long-term thyroid hormone replacement, as well as stage 1A ductal carcinoma of the breast diagnosed at age 42. In the same year she was diagnosed with cancer, she had a mastectomy and was given 3 years of adjuvant tamoxifen with no recurrence of disease in follow-up. During this treatment she underwent menopause. At age 50, a screening BMD showed a T score of −2.5 at the lumbar spine (LS), −2.5 at the femoral neck, and −2.6 at the total hip (TH), and she was offered alendronate 70 mg orally once weekly. Using these values, her Fracture Risk Assessment Tool score was calculated to be 1.1% for a hip fracture and 5.0% for a major osteoporotic fracture. For uncertain reason, after 1 month, she was switched to denosumab 60 mg injection every 6 months. She was on no other medications prior to her fractures.

While on denosumab, she experienced no fragility fractures, and a follow-up BMD taken 14 months after her screening BMD (10 months after denosumab initiation) showed a 6.8% increase in BMD at the LS and a 4.7% increase at the TH (T-scores −2.0 and −2.4, respectively). After a total of 5 denosumab injections given at 6-month intervals, the medication was discontinued on the belief that she had taken it for long enough, and there was no transition to another antiresorptive agent. Follow-up bone densitometry 10 months after the last denosumab dose (27 months after her second BMD) showed a nonsignificant reported change of −1.7% at the LS and +0.8% at the TH.

Eleven months after the final denosumab dose, the patient had sudden onset of severe back pain while walking. The pain caused significant impairment of mobility to the point where gait aids and a spine brace were needed for any standing or mobilization.

## Diagnostic Assessment

**Figure 1.**
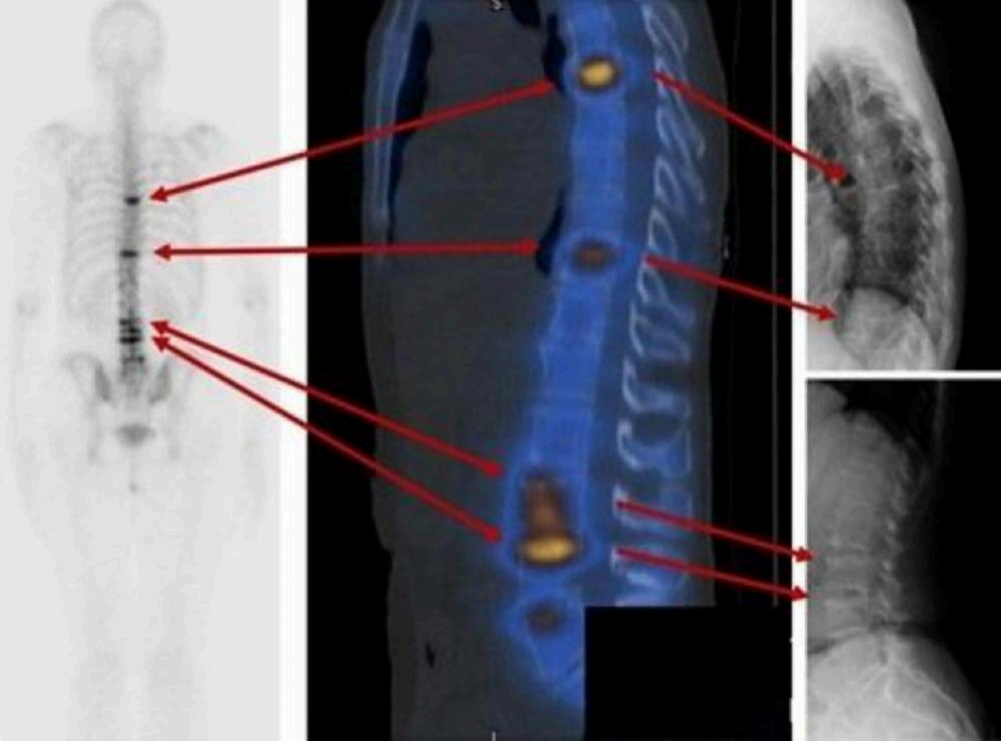
Nuclear medicine bone scan with single-photon emission computed tomography/computed tomography imaging and lateral spine X-ray. Horizontal bands of increased activity at T7, T11, L3, and L4 due to acute fractures.

**Figure 2.**
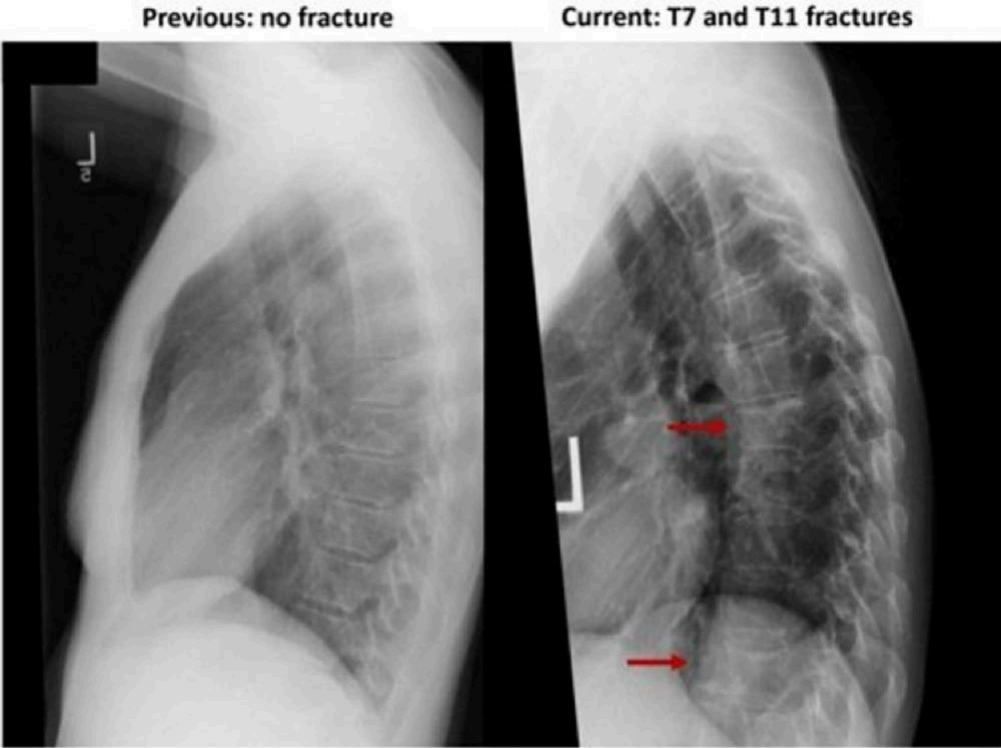
Thoracic spine X-rays, 13 years prior to and immediately following acute back pain.

**Figure 3.**
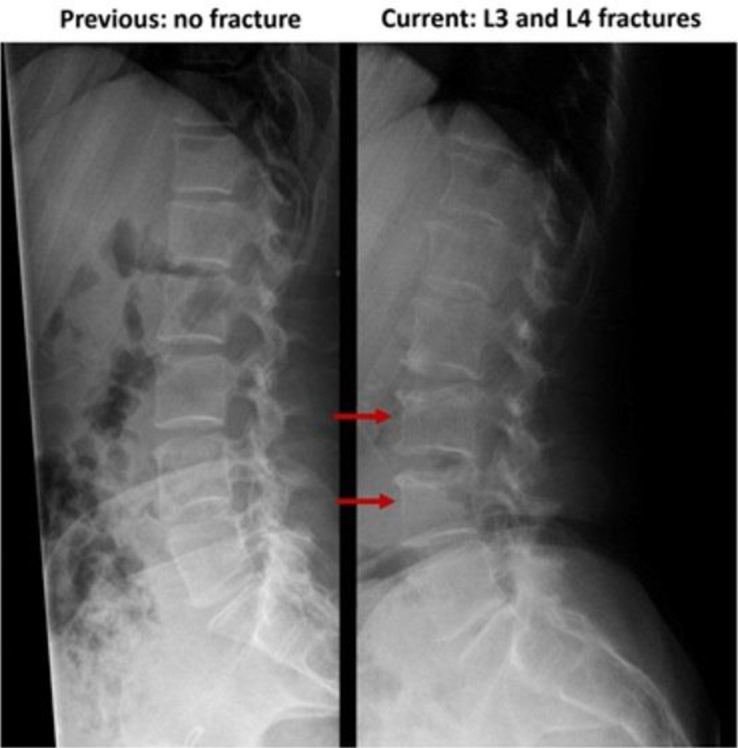
Lumbar spine X-rays, 10 years prior to and immediately following acute back pain.

Spine X-ray and bone scan showed acute compression fractures at the T7, T11, L3, and L4 vertebral bodies (Figs. 1, 2, and 3), all of which were new from previous X-rays. Bloodwork demonstrated an elevated collagen crosslinked C-terminal telopeptide (CTX) at 1341 ng/L (0-400 ng/L), suggesting accelerated bone resorption. At the time of her fractures, she had a height of 165.5 cm and a weight of 59.4 kg, with a body mass index of 21.7.

Bloodwork drawn 2 months prior to her fractures had shown calcium 2.54 mmol/L (2.10-2.60 mmol/L; 10.19 mg/dL), TSH 0.11 mIU/L (0.2-4.0 mIU/L), free thyroxine 23.4 pmol/L (10.0-25.0 pmol/L), creatinine 72 umol/L (40-100 umol/L; 0.81 mg/dL), estimated glomerular filtration rate 82 mL/min/1.73 m^2^ (≥60). Prior serum alkaline phosphatase was normal. Her TSH 6 months prior to her fractures was normal at 1.09, and she was clinically euthyroid while on levothyroxine 125 mcg daily at the time of her fracture. There was no evidence of recurrence of breast cancer from clinical examination or imaging including annual mammogram either at the time of vertebral fracture or on follow-up.

## Treatment

After clinical assessment, the patient was given risedronate 35 mg orally once weekly, which she tolerated well.

## Outcome and Follow-up

Follow-up CTX was 873 ng/L at 3 months and 464 ng/L at 6 months, where it remained stable over the next 2 years while on this therapy (Table 1). She experienced no further fractures and improved from a pain and mobility perspective. After 2 years of risedronate therapy, repeat bone densitometry showed nonsignificant changes of +2.9% at the LS and −1.5% at the TH (T-scores of −1.9 and −2.4, respectively). She has chosen to continue with bisphosphonate for an additional 3 years in light of her fracture experience.

**Table 1.** Collagen crosslinked C-telopeptide levels at various times after starting risedronate

	Pretreatment	3 months	6 months	9 months	16 months	20 months	22 months
Collagen crosslinked C-telopeptide (0-400 ng/L)	1341	873	464	572	406	468	458

## Discussion

### Theoretical Mechanisms of Denosumab Discontinuation-rebound

It has been observed that, after discontinuation of denosumab treatment, bone turnover markers rapidly rise and bone biopsy demonstrates increases in osteoclast number, osteoclast surface, and eroded bone surface with a simultaneous decrease in viable osteocytes and bone formation indices [6]. The pathophysiology of this increase in bone turnover is uncertain, and a proposed mechanism is that because denosumab does not induce osteoclast apoptosis, there is accumulation of dormant osteoclast precursors during the treatment period such that withdrawal of nuclear factor kappa B ligand inhibition results in their activation and subsequent increase in bone resorption [6, 7]. Another hypothesis revolves around the dysregulation of the Wnt signal transduction inhibitors sclerostin and Dickkopf-1 following treatment cessation, leading to increased bone turnover [6].

### Utility of Bisphosphonates in Prevention of Denosumab Discontinuation-rebound Losses

Small, short, randomized trials have suggested that the use of oral or intravenous bisphosphonates may help to reduce the loss of BMD following denosumab discontinuation. A study looking at zoledronic acid use in women previously treated with denosumab for 4.6 years found that there was still a 4% to 5% decrease in BMD at 12 months [8]. In comparison, women who did not receive zoledronic acid retained only 10% to 20% of their BMD gains over the same period [8]. Interestingly, those with **≤**6 injections of denosumab had a +1% gain in BMD after 12 months of zoledronic acid while those with >6 injections had a −7% loss, suggesting that a longer duration of denosumab therapy is associated with decreased protection from bisphosphonates after discontinuation [9]. A retrospective chart review of nearly 800 patients who had received at least 2 injections of denosumab found that the use of bisphosphonates after denosumab discontinuation reduced vertebral fractures by 93% [10]. Additionally, exposure to bisphosphonates prior to initiating denosumab was protective and reduced vertebral fractures by 78% [10].

Strengths of this case report include the fact that the patient was relatively young with no prior fragility fractures and had few comorbidities or medications to confound the clinical picture such that the link to denosumab discontinuation seems clear. The severity of her symptoms and her associated disability also highlight the impact that these events can have on patients’ lives. Potential limitations include the fact that we cannot prove a causal relationship between the discontinuation of denosumab and the patient's vertebral fractures and her acute fractures would have contributed to her elevated CTX, although a relationship to denosumab is likely given the time course and degree CTX of elevation.

## Learning Points

- Abrupt denosumab discontinuation is associated with multiple vertebral fractures.
- Bisphosphonates are the main therapy for the prevention/treatment of this phenomenon.
- When starting patients on denosumab antifracture therapy, education around the risk of unplanned discontinuation should be provided and post-discontinuation bisphosphonate strongly considered.

## Data Availability

Original data generated and analyzed during this study are included in this published article.
